# A mini-review of medical question answering for Bangla: the methodological landscape and cross-lingual perspectives

**DOI:** 10.3389/fdgth.2026.1856734

**Published:** 2026-10-14

**Authors:** Jannatul Somiya Mahmud, Khadija Akter Ratri, Md. Hamid Hosen, Md Kishor Morol, Liew Tze Hui, Dip Nandi, Abdullah Al Jubair, Mashiour Rahman

**Affiliations:** 1 Department of Computer Science and Engineering, BRAC University, Dhaka, Bangladesh; 2 Department of Computer Science and Engineering, University of Asia Pacific, Dhaka, Bangladesh; 3 Department of Computer Science and Engineering, East Delta University, Chittagong, Bangladesh; 4 ELITE Research Lab, LLC, Queens, New York, NY, United States; 5 Faculty of Information Science and Technology, Multimedia University, Melaka, Malaysia; 6 Department of CSE, American International University-Bangladesh, Dhaka, Bangladesh

**Keywords:** Bangla NLP, healthcare AI, large language models, medical question answering, retrieval-augmented generation

## Abstract

Large language models (LLMs) have advanced medical question answering (QA) significantly, yet low-resource languages such as Bangla remain critically underserved. This review examines the current landscape of LLM-based medical QA with a primary focus on Bangla, analyzing 15 representative studies (2022–2026), including English and multilingual works reviewed to contextualize findings for Bangla. We examine methodological approaches, datasets, and evaluation frameworks across diverse paradigms, including transformer architectures, retrieval-augmented generation (RAG), prompt engineering, and parameter-efficient fine-tuning, to identify pathways toward reliable and linguistically inclusive medical AI. The findings reveal a persistent performance gap driven by large-scale domain-specific pretraining and standardized benchmarks in English. Persistent challenges include dataset scarcity, heavy reliance on translated corpora, inconsistent evaluation practices, and a lack of clinician-validated benchmarks. This review identifies these gaps and outlines a structured research roadmap emphasizing native-language corpus development, multimodal data integration, standardized benchmarking, and bias auditing as essential steps toward building reliable and linguistically inclusive medical QA systems for Bangla healthcare.

## 1 Introduction

The emergence of large language models (LLMs) has brought about a paradigm shift in natural language processing, enabling state-of-the-art performance across diverse tasks, including contextual reasoning, generative text production, and domain-specific inference (1–3). This progress is particularly evident in healthcare, where LLM-enhanced systems support diagnostic precision and treatment planning (4–7). Among clinical NLP applications, medical question answering (QA) is particularly promising (8–10), bridging specialized medical expertise and public health accessibility (11), improving health outcomes through early symptom recognition, accessible health information, and increased health awareness (12–15). However, substantial performance gains have been achieved primarily for English (16–18), where extensive annotated datasets enable robust medical knowledge encoding (19, 20). Yet, this progress does not reflect the linguistic diversity of global healthcare needs. Bangla, despite being one of the world’s most widely spoken languages, remains critically underserved in medical NLP research, facing significant data scarcity and fragmented methodological development across multiple dimensions (21–23).

As LLMs become increasingly integrated into everyday life, people are turning to AI-powered systems for medical guidance (24, 25). Reliability and linguistic inclusivity become pressing public health concerns. Yet despite growing interest in medical QA, the literature remains English-centric, with Bangla receiving only fragmented attention. Inconsistent evaluation frameworks, datasets of vastly different scales, and absent shared benchmarks make cross-study comparison unreliable. The comparative effectiveness of key approaches, including fine-tuning vs. RAG and zero-shot vs. instruction tuning, remains empirically unclear (26). Promising techniques such as agentic RAG and parameter-efficient fine-tuning have not yet been consolidated for Bangla medical QA (27). This gap motivates a structured review to consolidate existing evidence to ensure that AI-powered medical guidance is accurate, reliable, and linguistically inclusive for all users, not just those in high-resource language settings.

This review presents a focused synthesis of LLM-based approaches to medical QA with primary emphasis on Bangla as a critically underserved case. Our primary research question is: *What is the current state of LLM methodologies for Bangla medical question answering, and what are the most promising pathways toward reliable and linguistically inclusive systems?* To address this, we analyze existing methodologies, benchmark datasets, evaluation frameworks, and challenges with the following contributions:

- A comparative analysis of Bangla medical QA systems against English-language benchmarks.
- A taxonomy of methodological approaches used in Bangla and related medical NLP and QA research.
- Evaluation of existing datasets, metrics, and key challenges specific to Bangla medical QA.
- Identification of research pathways toward reliable and linguistically inclusive medical AI for Bangla.

### 1.1 Literature search and study selection

A structured literature search was conducted to identify representative studies on large language model (LLM)-based medical question answering (QA), with particular emphasis on Bangla, alongside English and multilingual studies providing relevant comparative context. The search was designed for a focused mini-review rather than a formal systematic review or meta-analysis; therefore, the aim was to identify representative and methodologically relevant studies rather than exhaustively capture all available literature.

Literature published between 2022 and 2026 was considered. Searches were conducted across IEEE Xplore, ACM Digital Library, SpringerLink, PubMed, and Google Scholar using combinations of terms related to “large language model”, “LLM’,‘medical question answering”, “medical QA”, “Bangla”, “Bengali”, “multilingual”, “low-resource language”, and “multimodal”. Boolean operators were used to combine these concepts. The initial literature assessment also considered relevant preprints, particularly arXiv papers, as well as studies on other low-resource languages and multimodal medical QA to capture recent and relevant developments.

Studies were considered for detailed synthesis when they investigated LLMs or transformer-based language models in medical or healthcare language-processing tasks, addressed medical QA or closely related medical NLP tasks, and provided sufficient information on datasets, methodology, prompting or training strategies, and/or evaluation. Related tasks, such as medical named-entity recognition and summarization, were retained only when they provided relevant evidence for language-specific model behavior or evaluation. Studies were excluded if they addressed unrelated Medical NLP tasks, lacked evaluation detail, or were not peer-reviewed.

Ultimately, 15 representative studies were selected for detailed synthesis. The review focused primarily on Bangla within the low-resource language literature, while English-language studies were included for comparison and two multilingual studies were included to provide broader cross-lingual context. Selected multimodal studies were also included where relevant to medical QA. The final selection covered transformer fine-tuning, prompt-based methods, retrieval-augmented generation (RAG), parameter-efficient adaptation, multilingual, and multimodal approaches.

## 2 Evolution of medical question answering systems

Medical QA systems have evolved from rule-based methods to neural architectures and retrieval-augmented large language models. In Bangla contexts, this transition reflects both technological advancement and persistent low-resource challenges. The field now emphasizes multilingual benchmarking and parameter-efficient fine-tuning. Recent studies increasingly explore multilingual medical QA; however, this research focuses specifically on English and Bangla. English serves as a high-resource benchmark, while Bangla represents a low-resource setting, enabling a more focused and meaningful comparison of resource disparities and model performance.

To organize the methodological diversity of the reviewed literature independently of publication date, the included studies are additionally classified by the mechanism that produces their answers at inference time; for example, a hand-written rule, a fine-tuned classifier, a frozen model conditioned only by its prompt, or a generator conditioned on retrieved documents. Where a study combines mechanisms, it is assigned to the one its own ablation or evaluation credits with the principal performance gain. Related medical NLP tasks, including named-entity recognition and summarization, are retained in this classification only where they provide evidence of language-specific model behavior transferable to medical QA. The resulting taxonomy, covering eight paradigms and their practical implications for Bangla, is presented in Table 1.

**Table 1:** Taxonomy of methodological approaches in medical QA and related medical NLP.

Methodological paradigm	Defining characteristic	Learning requirement	Language and resource considerations	Representative studies
Rule-based/symbolic	Hand-crafted rules, lexicons, and structured queries	No model training	Highly language-specific resources	Mandal et al. (28)
Supervised neural/hybrid	Neural models with task-specific features or embeddings	Requires labelled data	Depends on language-specific preprocessing and data	Tasnia and Hoque (29)
Transformer fine-tuning	Pretrained transformers adapted to a medical task	Requires task-specific fine-tuning data	Benefits from target-language pretrained models	Khan et al. (30, 31)
Prompt-based LLM inference	Zero-/few-shot inference through prompt design	No parameter updates	Depends on pretrained model’s language coverage	Abrar et al. (32); Hemel et al. (33)
Parameter-efficient adaptation	Adapts pretrained models using lightweight parameter updates	Requires task-specific data	Lower adaptation cost; base-model language coverage remains important	Sen et al. (34)
Retrieval-augmented generation	Retrieves external knowledge to support generation	Depends on retrieval framework; LLM may remain frozen	Requires suitable domain/language knowledge sources	Shi et al. (35); Sohn et al. (36); Alonso et al. (17)
Multi-task learning	Joint learning of multiple related tasks	Requires supervision for multiple tasks	Requires compatible task annotations	Pattnayak et al. (37)
Multimodal QA	Combines text with medical images or other modalities	Requires aligned multimodal data	Depends on paired multimodal resources	Saeed (38)

### 2.1 Rule-based and classical machine learning approaches

Early Bangla medical QA systems were primarily rule-based and database-driven. For instance, Mandal et al. (28) proposed XBLQPS, which combined tokenization, root extraction, POS tagging, and a simplified LESK algorithm for word sense disambiguation, mapping linguistic features into predefined rules for structured query retrieval.

Later, research shifted toward statistical and classical machine learning approaches. Tasnia and Hoque (29) developed datasets (BnMedText, BnMedQA) and applied preprocessing, feature extraction (TF-IDF, Word2Vec, FastText), and classifiers such as SVM and Random Forest, alongside a hybrid CNN–BiLSTM model (MeQANet). Similarly, Khan et al. (30) introduced Bangla-HealthNER using BERT-based models for entity recognition. Despite this progress, these methods remained limited by handcrafted features and constrained contextual understanding.

### 2.2 Neural and transformer-based architectures

The introduction of transformer-based models significantly enhanced contextual representation. Khan et al. (31) curated BanglaCHQ-Summ and fine-tuned transformer models, including BanglaT5, mT5, and mBART, for medical summarization. Abrar et al. (32) expanded experimentation by evaluating zero-shot and instruction-tuned large language models (LLMs) without task-specific fine-tuning, relying on iterative prompt refinement to generate concise Bangla summaries.

Parameter-efficient adaptation emerged as a critical methodological advancement. Sen et al. (34) applied LoRA-based fine-tuning to a Gemma-2B model, combining OCR-based prescription extraction (PyTesseract), GloVe embeddings, CNN/RNN encoders, and BiDAF attention before domain adaptation. In English contexts, Pattnayak et al. (37) implemented a multi-task learning framework using ClinicalBERT with shared encoders and dual task-specific heads for answer extraction and entity classification. Their pipeline incorporated WordPiece tokenization, UMLS-based NER, and weighted loss optimization. Hemel et al. (33) explored structured prompt engineering strategies, including zero-shot, few-shot, leap prompting, and role-play, combined with 4-bit model quantization to improve computational efficiency. Collectively, these studies reflect a transition toward contextual transformers, prompt engineering, and parameter-efficient adaptation.

### 2.3 Retrieval-augmented and large-scale LLM frameworks

Recent methodologies increasingly integrate retrieval mechanisms and large-scale benchmarking. Shi et al. (35) proposed MKRAG, combining two-stage retrieval with knowledge injection through in-context learning. Sohn et al. (36) introduced RAG2, which generated chain-of-thought rationales for query formulation, balanced retrieval across biomedical corpora, and trained a perplexity-guided filtering model to refine retrieved evidence. Large-scale multilingual datasets further advanced fine-tuning paradigms. Alonso et al. (17) combined gold knowledge grounding with automated retrieval (BM25, MedCPT, reciprocal rank fusion) before fine-tuning multilingual models.

## 3 Literature synthesis

Table 2 summarizes 15 representative studies on medical question answering across English, Bangla, and multilingual. It compares datasets, methodologies, model architectures, evaluation metrics, and limitations.

**Table 2:** Comparative summary of representative medical question answering studies across English, Bangla, and multilingual settings.

Author (Year)	Language	Dataset	Model	Metrics	Key Findings	Limitations
Pattnayak et al. (37) (2025)	English	emrQA	ClinicalBERT	Answer extraction F1: 81.7%; classification accuracy: 90.7%	MTL boosts extraction F1 by 2.2% and classification accuracy by 6.2% over single-task	No multilingual support; span boundary errors; ambiguous answers.
Shi et al. (35) (2025)	English	MedQA-USMLE	Vicuna-7B	Accuracy: 44.46% (Vanilla Vicuna), 48.54% (MKRAG)	RAG improves accuracy by 4.08% without fine-tuning; Contriever outperforms SapBERT.	Context length limits; irrelevant facts may be included.
Abrar et al. (32) (2024)	Bangla	BanglaCHQ-Summ	Zero-shot LLMs, Bangla T5	ROUGE-1: Mixtral 51.36%, Bangla T5 50.05%; ROUGE-2: Bangla T5 29.11%; ROUGE-L: Mixtral 49.17%	Zero-shot LLMs rival fine-tuned Bangla T5 on R1/RL; Bangla T5 leads on R2.	Phonetic variation in Bangla reduces ROUGE scores; lack of task-specific training limits bi-gram accuracy.
Hemel et al. (33) (2024)	Bangla	Bangla Medical Dataset (659 samples)	Llama3-8b, Mistral-7b, Gemma-7b, Phi-3-mini-4k, Qwen2-7b, GPT-4-Turbo	Zero-shot F1: 38.2% (GPT-4-Turbo); mix prompt F1: 86.1% (GPT-4-Turbo)	Mix prompting dramatically improves F1; Qwen was most resource-efficient.	Dataset class imbalance, gender/organ biases; manual prompt engineering.
Bernal-Utrera et al. (39) (2026)	English	Breast cancer CPG-based questions	ChatGPT-4	Accuracy: 7% reasonably accurate (1/14)	ChatGPT-4 showed only 7% accuracy for breast cancer exercise counseling.	Single LLM evaluated; limited to the breast cancer domain; opacity of training data.
Sohn et al. (36) (2025)	English	MedQA, MedMCQA, MMLU-Med	Llama-3-8B, Meerkat-7B, GPT-4o + RAG2	Accuracy gains: Llama-3 6.1%, Meerkat 3.8%, GPT-4o 0.9%	RAG2 improves accuracy by up to 6.1% via rationale queries, balanced retrieval, and filtering.	Not tested on general domains; incorrect rationales degrade retrieval.
Arias-Duart et al. (40) (2025)	English	CareQA	12 open LLMs	Weak correlation (r<0.3) between open and closed benchmarks	Weak correlation between open-ended and closed-ended benchmarks; Relaxed Perplexity proposed as factuality metric.	No bias assessment or expert medical review; limited model set.
Wang et al. (41) (2025)	English	COVID-QA, Medical Meadow MedQA, PubMedQA, MedMCQA	LLaMA-7B, Mistral, Zephyr-7B-Alpha, Vicuna-7B, WizardLM-7B, StableBeluga-7B	WSE_C AUROC: 0.6235 (overall); accuracy improvement up to 6.36%	WSE improved model accuracy by up to 6.36% without additional fine-tuning.	Requires token likelihoods; incompatible with black-box APIs; cannot fully capture model uncertainty.
Tasnia and Hoque (29) (2024)	Bangla	BnMedQA, BnMedText	MeQANet (CNN + BiLSTM)	F1: 85%	CNN + BiLSTM hybrid outperforms SVM and BERT for Bangla medical QA.	Class imbalance; translated data quality issues; only 8 specialties.
Mandal et al. (28) (2022)	Bangla	Modified Bangla WordNet + Healthcare DB	Rule-based XBLQPS	Accuracy: 70%	LESK algorithm resolves Bangla lexical ambiguity; no SQL knowledge needed.	Simplified WSD; only structured data; small dataset.
Khan et al. (30) (2023)	Bangla	Bangla-HealthNER (31,783 samples)	BanglaBERT, mBERT, BanglishBERT, GPT-3.5 (zero-shot)	F1 (%): 54.74±0.34, 54.98±0.82, 56.13±0.75, 15.51	Language-specific + code-switched models outperform multilingual ones for NER.	IOB tagging struggles with split multi-word entities.
Khan et al. (31) (2023)	Bangla	BanglaCHQ-Summ (2,350 pairs)	BanglaT5	ROUGE-L: 48.35%; BERTScore: 89.91%	First Bangla CHQ dataset; language-specific models beat multilingual models for summarization.	Modest dataset size; high compute requirements.
Sen et al. (34) (2024)	Bangla	300 Medical QA pairs (42 disease categories)	Gemma 2B + LoRA	Qualitative only	First Bangla medical QA dataset; LoRA fine-tuning is effective for domain adaptation.	Only 300 samples; no baseline comparison; text-only.
Saeed (38) (2024)	Multilingual: English, Chinese, Spanish	MEDIQA-M3G (dermatology)	VGG16-CNN-SVM + ViT-CLIP	3rd rank (ZH/ES), 7th (EN)	Weak supervision with CLIP selection effective; translation improves BERTScore.	Small dataset (842); machine translation errors; no clinical validation.
Alonso et al. (17) (2024)	Multilingual: English, Spanish, French, Italian	Antidote CasiMédicos / MedExpQA	Mistral-7B (fine-tuned)	Accuracy 91.7% (gold grounding)	Gold explanations produce 91.7% accuracy; fine-tuning reduces RAG dependence.	No GPT-4 fine-tuning; MedCorp English-only; accuracy metric limitations.

### 3.1 Bangla medical QA

Bangla medical QA research has progressed rapidly, yet persistent structural constraints remain. The most critical is dataset scarcity: existing corpora are either modest in scale, such as the 4,500 QA pairs used by Tasnia and Hoque (29) or the 659 patient-symptom pairs used by Hemel et al. (33), or rely heavily on machine translation from English sources. Classifiers trained on translated data tend to collapse on semantically broad categories like General Medicine, where cross-domain overlap is high, while performing reliably on specialties with distinctive vocabulary like Gynecology or ENT.

The fine-tuning vs. prompting debate also varies across medical NLP tasks. In structured tasks such as named entity recognition, BanglishBERT achieved the highest F1 (56.13±0.75%), followed by mBERT (54.98±0.82%) and BanglaBERT (54.74±0.34%) on informal Bangla clinical text, whereas zero-shot GPT-3.5 achieved an F1 of 15.51% on the Bangla-HealthNER dataset (30). These results should not be interpreted as a direct comparison with open-ended medical QA. In a separate study on specialty recommendation, Hemel et al. (33) reported an F1 of 38.2% for zero-shot GPT-4-Turbo and 86.1% with mixed prompting on the same 659-pair dataset. Overall, these findings illustrate task-specific differences between fine-tuned and prompted approaches rather than providing a direct cross-study comparison.

### 3.2 English medical QA

English medical QA research has advanced along sophisticated architectural, retrieval, and evaluation dimensions. Models like the ClinicalBERT variant by Pattnayak et al. (37) demonstrate that joint training for answer extraction and medical categorisation yields mutual performance gains. Retrieval-augmented generation has emerged as the primary strategy for bridging LLMs’ knowledge gaps; while injecting external facts improves performance (35), retrieval quality matters more than quantity, as irrelevant context actively degrades accuracy (36), shifting focus from what to retrieve toward how to filter.

Uncertainty quantification remains neglected despite its clinical significance. Wang et al. (41) show that selecting lowest-uncertainty responses improves accuracy without architectural changes, suggesting that knowing when not to answer is as clinically valuable as answering correctly. Most consequentially, strong benchmark scores do not translate to trustworthy responses in nuanced clinical contexts (39), raising fundamental questions about whether current evaluation frameworks adequately prepare systems for real-world deployment.

## 4 Comparative analysis and discussion

Across the reviewed literature, English-language medical QA demonstrates a broader and more established methodological landscape than Bangla, particularly in benchmark availability, model adaptation, and evaluation. Bangla research, in contrast, is characterized by a smaller but increasingly diverse body of work spanning supervised fine-tuning, prompting, and task-specific adaptation. The comparison therefore suggests a difference not only in model performance, but also in the maturity and breadth of the surrounding research ecosystem. Because the reviewed studies employ different datasets, task formulations, and evaluation protocols, numerical results are interpreted primarily within individual study settings rather than used to establish a universal ranking across models.

English-language medical QA benefits from large-scale biomedical resources and established evaluation benchmarks, enabling systematic investigation of model adaptation and retrieval-based methods. Within individual studies, multi-task learning improved answer-extraction F1 by 2.2% and classification accuracy by 6.2% over ClinicalBERT fine-tuned for QA on emrQA (37). Similarly, rationale-guided retrieval augmentation improved accuracy by up to 6.1% across MedQA, MedMCQA, and MMLU-Med (36). These findings illustrate a broader trend toward combining pretrained language models with task-specific supervision and external knowledge retrieval rather than relying solely on model scale. Methods such as MKRAG (35) and RAG2 (36) further reflect this shift toward retrieval-based and knowledge-grounded medical QA.

Bangla medical QA research shows a similar movement toward task-specific adaptation, although the evidence base remains smaller. BnMedQA contains approximately 4,500 QA pairs (29), while BanglaCHQ-Summ contains 2,350 summarization pairs (31). Within their respective evaluation settings, MeQANet achieves an F1 score of 0.85 for eight-way specialty classification on BnMedQA (29), while mixed prompting of GPT-4-Turbo achieves an F1 score of 86.1% on a separate Bangla specialty-recommendation dataset (33). Although these results use the same metric, differences in task definition, datasets, and label spaces prevent direct performance ranking. Nevertheless, the studies demonstrate that Bangla medical NLP is progressing beyond basic language modeling toward task-specific medical reasoning and decision-support applications.

A notable distinction between the two research ecosystems lies in the role of evaluation resources. English medical QA has benefited from widely used benchmarks that facilitate repeated evaluation of emerging models and methods. In Bangla, benchmark development is more recent and fragmented, resulting in greater variation in task formulation and evaluation practices. This difference affects not only the comparability of reported results but also the ability to establish reproducible baselines and measure progress across successive studies. The development of larger, carefully curated Bangla medical QA benchmarks would therefore provide an important foundation for more rigorous model evaluation.

The asymmetry described above is structural rather than incidental, as Figure 1 makes clear by contrasting the high-resource English and low-resource Bangla medical QA ecosystems. Although both environments share core development layers, including datasets, LLM-based modeling, evaluation, and clinical validation, their maturity differs substantially. English research benefits from established benchmarks and evaluation practices, whereas Bangla research remains constrained by limited resources, heterogeneous evaluation, and comparatively limited clinical integration. The priority for Bangla is therefore not simply to replicate English models, but to develop robust language-specific resources and evaluation settings that can support meaningful assessment of increasingly capable LLM-based medical QA systems.

**Figure 1:**
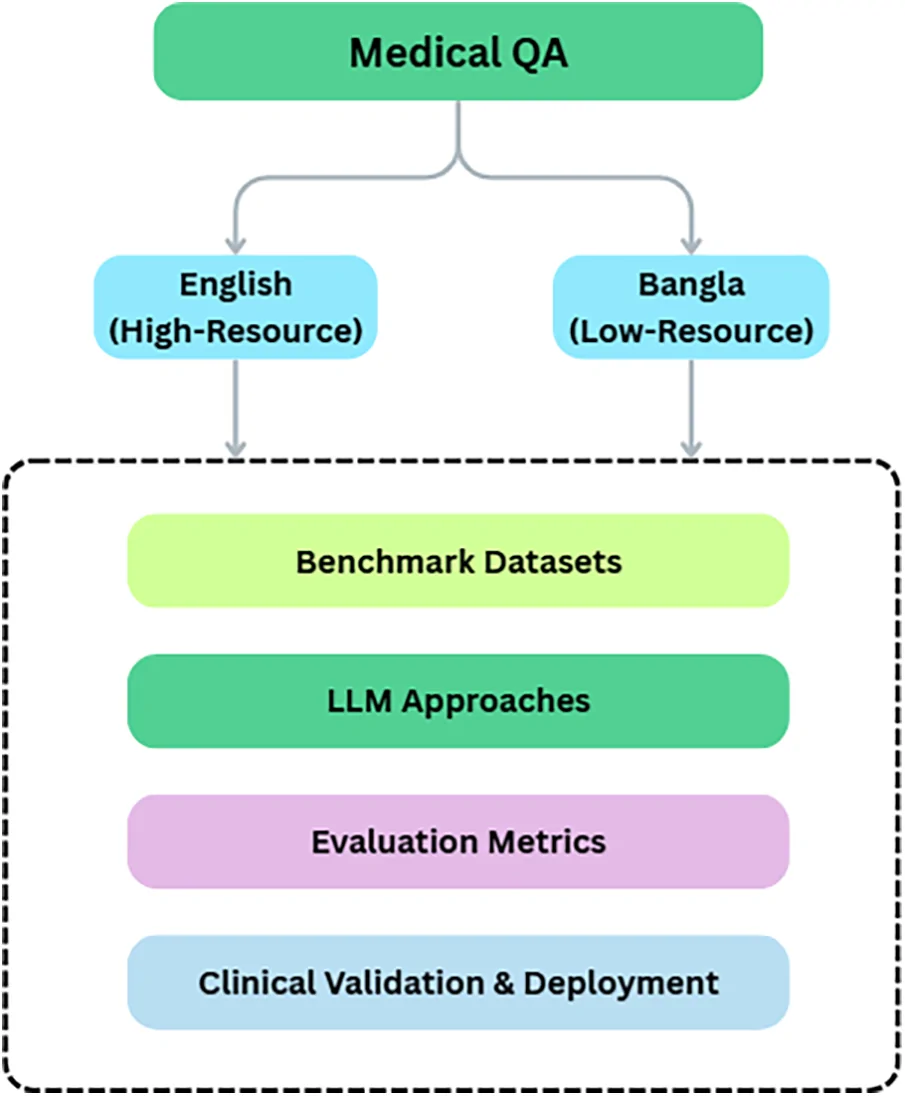
Comparative ecosystem of medical QA across high- and low-resource languages.

## 5 Limitations and future works

The reviewed studies reveal several critical limitations. The most persistent challenge is dataset scarcity: existing datasets are too small for training modern architectures, and no Bangla symptom-based diagnostic QA dataset exists. The few available Bangla medical QA datasets heavily rely on translated English sources, introducing semantic drift and cultural misalignment, and none include clinician-validated ground truth. Datasets are also predominantly text-only, failing to capture multimodal clinical realities such as medical imaging. Linguistic complexity poses an additional barrier, as Bangla’s rich morphology, dialectal variation, and code-switching remain poorly handled by existing models. Model-level limitations are equally significant: LLMs trained primarily on English corpora exhibit biases such as gender-based misclassification and keyword-driven organ association rather than nuanced clinical reasoning. Finally, the evaluation methodology remains underdeveloped; ROUGE and BERTScore dominate Bangla QA evaluations, yet they correlate poorly with clinical utility, and no standardized benchmarks or clinical validation exist across the literature.

Future research should prioritize constructing the first Bangla symptom-based diagnostic QA dataset with clinician-validated ground truth, alongside larger expert-annotated corpora incorporating dialectal variation, code-mixed text, and multimodal data such as medical images, directly addressing current dataset gaps. Domain-specific pretraining on native Bangla medical text is essential, with parameter-efficient methods like LoRA and QLoRA offering practical solutions for resource-constrained settings. RAG pipelines should be rebuilt around native-language knowledge bases to reduce dependence on English corpora. Standardized benchmarks derived from sources excluded from model pretraining would enable reliable cross-study comparison. Finally, bias auditing and lightweight deployment strategies should be prerequisites for any clinical deployment.

## 6 Conclusion

This mini review provides a focused, structured synthesis of LLM-based medical question answering (QA) systems with a comparison between English and Bangla. The analysis shows a clear evolution from rule-based and classical machine learning approaches to transformer-based architectures and retrieval-augmented large language models. Despite these advancements, a significant gap persists between the two ecosystems. English medical QA systems benefit from large biomedical corpora, standardized benchmarks, retrieval-grounded architectures, and rigorous evaluation practices. In contrast, Bangla medical QA remains constrained by limited dataset scale, dependence on translated resources, insufficient domain-specific pretraining, and inconsistent evaluation frameworks. Evidence suggests that retrieval quality and access to curated native-language medical knowledge bases play a more critical role than model scale alone. Parameter-efficient fine-tuning techniques such as LoRA and QLoRA, together with emerging agentic retrieval-augmented generation frameworks, therefore represent promising and computationally feasible pathways for advancing medical QA in resource-constrained environments. However, benchmark performance alone is insufficient for real-world deployment; physician validation, bias assessment, and uncertainty calibration remain essential. Future progress depends on developing expert-annotated Bangla datasets, establishing standardized evaluation protocols, and integrating clinically grounded validation frameworks.

## References

[1] Mei L Yao J Ge Y Wang Y Bi B Cai Y, et al. Data from: A survey of context engineering for large language models. (2025). 10.48550/arXiv.2507.13334

[2] Lee S Sim W Shin D Seo W Park J Lee S, et al. Reasoning abilities of large language models: in-depth analysis on the abstraction and reasoning corpus. ACM Trans Intell Syst Technol. (2025) 16:1–52. 10.1145/3712701

[3] Xiong S Payani A Kompella R Fekri F. Large language models can learn temporal reasoning. In: *Proceedings of the 62nd annual meeting of the association for computational linguistics (volume 1: long papers)* (Association for Computational Linguistics) (2024), 10452–70. 10.18653/v1/2024.acl-long.563 PMC12007664 40255468

[4] Adib SES Sani AA Esham EA Abrar A Chowdhury TM. Data from: Assessing large language models for medical QA: zero-shot and LLM-as-a-judge evaluation. (2026). 10.48550/arXiv.2602.14564

[5] Maity S Saikia MJ. Large language models in healthcare and medical applications: a review. Bioengineering. (2025) 12:631. 10.3390/bioengineering12060631 40564447 PMC12189880

[6] Singhal K Tu T Gottweis J Sayres R Wulczyn E Amin M, et al. Toward expert-level medical question answering with large language models. Nat Med. (2025) 31:943–50. 10.1038/s41591-024-03423-7 39779926 PMC11922739

[7] Busch F Hoffmann L Rueger C Van Dijk EHC Kader R Ortiz-Prado E, et al. Current applications and challenges in large language models for patient care: a systematic review. Commun Med. (2025) 5:26. 10.1038/s43856-024-00717-2 39838160 PMC11751060

[8] Sait ARW Alkhurayyif Y. Explainable bilingual medical-question-answering model using ensemble learning technique. Electronics. (2025) 14:4128. 10.3390/electronics14204128

[9] Zafar A Sahoo SK Bhardawaj H Das A Ekbal A. KI-MAG: a knowledge-infused abstractive question answering system in medical domain. Neurocomputing. (2024) 571:127141. 10.1016/j.neucom.2023.127141

[10] Demirhan H Zadrozny W. Survey of multimodal medical question answering. BioMedInformatics. (2024) 4:50–74. 10.3390/biomedinformatics4010004

[11] Zhang S Song J. A chatbot based question and answer system for the auxiliary diagnosis of chronic diseases based on large language model. Sci Rep. (2024) 14:17118. 10.1038/s41598-024-67429-4 39054346 PMC11272932

[12] Faris H Habib M Faris M Elayan H Alomari A. An intelligent multimodal medical diagnosis system based on patients’ medical questions and structured symptoms for telemedicine. Inform Med Unlock. (2021) 23:100513. 10.1016/j.imu.2021.100513

[13] Kiru KU Pal SK Ahmad SS Kabir IM. Deep learning-enhanced medical system for disease prediction, medication recommendation, and intelligent question-answering based on patient symptomatology. In: *2024 IEEE 11th Uttar Pradesh section international conference on electrical, electronics and computer engineering (UPCON)* (2024), 1–8. 10.1109/UPCON62832.2024.10982945

[14] Luo X Li Y Xu J Zheng Z Ying F Huang G. AI in medical questionnaires: innovations, diagnosis, and implications. J Med Internet Res. (2025) 27. 10.2196/72398 PMC12401066 40789173

[15] Walker HL Ghani S Kuemmerli C Nebiker CA Müller BP Raptis DA, et al. Reliability of medical information provided by ChatGPT: assessment against clinical guidelines and patient information quality instrument. J Med Internet Res. (2023) 25:e47479. 10.2196/47479 37389908 PMC10365578

[16] Jin Y Chandra M Verma G Hu Y De Choudhury M Kumar S. Better to ask in english: cross-lingual evaluation of large language models for healthcare queries. In: *Proceedings of the ACM web conference 2024* (Association for Computing Machinery) (2024), WWW ’24, 2627–38. 10.1145/3589334.3645643

[17] Alonso I Oronoz M Agerri R. Medexpqa: multilingual benchmarking of large language models for medical question answering. Artif Intell Med. (2024) 155:102938. 10.1016/j.artmed.2024.102938 39121544

[18] Kumar GK Gehlot A Mullappilly SS Nandakumar K. MuCoT: multilingual contrastive training for question-answering in low-resource languages. In: Chakravarthi BR, Priyadharshini R, Madasamy AK, Krishnamurthy P, Sherly E, Mahesan S, editors. *Proceedings of the second workshop on speech and language technologies for dravidian languages* (Association for Computational Linguistics) (2022), 15–24. 10.18653/v1/2022.dravidianlangtech-1.3

[19] Eladio J Wu H. Data from: emrQA-msquad: a medical dataset structured with the SQuAD V2. 0 framework, enriched with emrQA medical information. (2024). 10.48550/arXiv.2404.12050

[20] Yue X Jimenez Gutierrez B Sun H. Clinical reading comprehension: a thorough analysis of the emrQA dataset. In: *Proceedings of the 58th annual meeting of the association for computational linguistics* (Online: Association for Computational Linguistics) (2020), 4474–86. 10.18653/v1/2020.acl-main.410

[21] Hu S Oppong A Mogo E Collins C Occhini G Barford A, et al. Natural language processing technologies for public health in Africa: scoping review. J Med Internet Res. (2025) 27:e68720. 10.2196/68720 40053738 PMC11923465

[22] Wang Y Lin M Hu Q Bao L Bai S Li Y. Large and small models for collaborative cross-lingual data augmentation in entity relationship extraction for low-resource languages. J King Saud Univ Comput Inform Sci. (2025) 37:56. 10.1007/s44443-025-00055-w

[23] Villena F Bravo-Marquez F Dunstan J. NLP modeling recommendations for restricted data availability in clinical settings. BMC Med Inform Decis Mak. (2025) 25:116. 10.1186/s12911-025-02948-2 40055634 PMC11889813

[24] Pal A Wangmo T Bharadia T Ahmed-Richards M Bhanderi MB Kachhadiya R, et al. Generative AI/LLMs for plain language medical information for patients, caregivers and general public: Opportunities, risks and ethics. Patient Prefer Adherence. (2025) 19:2227–49. 10.2147/PPA.S527922 40771655 PMC12325106

[25] Wang B Zheng Y Han X Kong L Xiao G Xiao Z, et al. A systematic literature review on integrating AI-powered smart glasses into digital health management for proactive healthcare solutions. NPJ Digit Med. (2025) 8:410. 10.1038/s41746-025-01715-x 40617964 PMC12228729

[26] Dong X Zhu W Wang H Chen X Qiu R Yin R, et al. Data from: Talk before you retrieve: agent-led discussions for better rag in medical QA. (2025). 10.48550/arXiv.2504.21252

[27] Shaitarova A Zaghir J Lavelli A Krauthammer M Rinaldi F. Exploring the latest highlights in medical natural language processing across multiple languages: a survey. Yearb Med Inform. (2023) 32:230–43. 10.1055/s-0043-1768726 38147865 PMC10751112

[28] Mandal KP Mukherjee P Chattopadhyay A Chakraborty B. Xblqps: an extended bengali language query processing system for e-healthcare domain. Int J Adv Comput Sci Appl. (2022) 13:502–16. 10.14569/IJACSA.2022.0130761

[29] Tasnia F Hoque MM. Meqanet: deep learning-based framework for classifying medical query answering in bengali. In: *Proceedings of the 2024 IEEE international conference on computing, applications and systems (COMPAS)* (2024), 1–7. 10.1109/COMPAS60761.2024.10796708

[30] Khan A Kamal F Nower N Ahmed T Ahmed S Chowdhury T. NERvous about my health: constructing a bengali medical named entity recognition dataset. In: *Findings of the association for computational linguistics: EMNLP 2023* (2023), 5768–74. 10.18653/v1/2023.findings-emnlp.383

[31] Khan A Kamal F Chowdhury MA Ahmed T Laskar MTR Ahmed S. BanglaCHQ-summ: an abstractive summarization dataset for medical queries in Bangla conversational speech. In: *Proceedings of the first workshop on bangla language processing (BLP-2023)* (Association for Computational Linguistics) (2023), 85–93. 10.18653/v1/2023.banglalp-1.10

[32] Abrar A Tabassum F Ahmed S. Performance evaluation of large language models in Bangla consumer health query summarization. *Proceedings of the 2024 27th international conference on computer and information technology (ICCIT)* (2024), 2748–53. 10.1109/ICCIT64611.2024.11022034

[33] Hemel RI Asef M Tamiti TI. Enhancing diagnostic accuracy in Bangla: LLM approaches to gender-based medical specialty prediction. In: *Proceedings of the 2024 27th international conference on computer and information technology (ICCIT)* (2024), 873–8.

[34] Sen A Dash SR Pradhan MR Parida S. Healthcare question answering system in bengali: a proposed model. J Theor Appl Inform Technol. (2024) 102:4958–71.

[35] Shi Y Xu S Yang T Liu Z Liu T Li X, et al. Mkrag: medical knowledge retrieval augmented generation for medical question answering. In: *AMIA … Annual Symposium proceedings. AMIA Symposium* (2025), 2024, 1011–20. PMC12099378 40417500

[36] Sohn J Park Y Yoon C Park S Hwang H Sung M, et al. Rationale-guided retrieval augmented generation for medical question answering. In: *Proceedings of the 2025 conference of the nations of the americas chapter of the association for computational linguistics: human language technologies (volume 1: long papers)* (Association for Computational Linguistics) (2025), 12739–53. 10.18653/v1/2025.naacl-long.635

[37] Pattnayak P Patel H Agarwal A Panda S Kumar B Kumar T. Clinical QA 2.0- multi-task learning for answer extraction and categorization. *2025 IEEE international conference on electro information technology (eIT)* (2025), 1–7.

[38] Saeed N. MediFact at MEDIQA-M3G 2024: medical question answering in dermatology with multimodal learning. In: *Proceedings of the 6th clinical natural language processing workshop* (Association for Computational Linguistics) (2024), 339–45.

[39] Bernal-Utrera C Bravo-Vázquez A Montero-Bancalero F Suárez-Vega A Casuso-Holgado M Anarte-Lazo E. Evaluation of ChatGPT accuracy and reliability in answering questions about exercise recommendations for breast cancer survivors. Physiotherapy. (2026) 130:101838. 10.1016/j.physio.2025.101838 41270301

[40] Arias-Duart A Martin-Torres PA Hinjos D Bernabeu-Perez P Ganzabal LU Mallo MG, et al. Automatic evaluation of healthcare LLMs beyond question-answering. In: *Proceedings of the 2025 conference of the nations of the americas chapter of the association for computational linguistics: human language technologies (volume 2: short papers)* (Association for Computational Linguistics) (2025), 108–30. 10.18653/v1/2025.naacl-short.10

[41] Wang Z Duan J Yuan C Chen Q Chen T Zhang Y, et al. Word-sequence entropy: towards uncertainty estimation in free-form medical question answering applications and beyond. Eng Appl Artif Intell. (2025) 139:109553. 10.1016/j.engappai.2024.109553

